# A Comparative Analysis of Arch Widths in Class I and Class II Malocclusion: Extraction vs. Non-extraction Treatment

**DOI:** 10.7759/cureus.57982

**Published:** 2024-04-10

**Authors:** Anusha Hegde, Lohith D, Shailaja A M, Ajith Geevee, Rohith M Srikant

**Affiliations:** 1 Orthodontics and Dentofacial Orthopaedics, Sri Dharmasthala Manjunatheshwara Dental College and Hospital, Sri Dharmasthala Manjunatheshwara University, Dharwad, IND; 2 Orthodontics and Dentofacial Orthopaedics, RajaRajeswari Dental College and Hospital, Bangalore, IND; 3 Orthodontics and Dentofacial Orthopaedics, Sri Siddhartha Dental college And Hospital, Tumkur, IND; 4 Orthodontics and Dentofacial Orthopaedics, Vinayaka Mission's Sankarachariyar Dental College, Salem, IND; 5 Orthodontics and Dentofacial Orthopaedics, Sumukh Dental Clinic, Bangalore, IND; 6 Orthodontics and Dentofacial Orthopaedics, Krittika Dental Clinic, Bangalore, IND; 7 Orthodontics and Dentofacial Orthopaedics, R. K. Dental Care, Bangalore, IND

**Keywords:** non-extraction treatment, extraction treatment, digital vernier caliper, class 2 malocclusion, class 1 malocclusion, arch width

## Abstract

Introduction: This study aimed to assess and compare dental arch widths in the anterior and posterior regions among patients undergoing extraction and non-extraction treatments for Class I and Class II malocclusions.

Materials and methodology: A total of 40 patients were selected, with 10 in each of the categorized groups based on malocclusion type and treatment status. Dental arch widths were meticulously measured using a digital Vernier caliper at the canine and molar regions to ensure precise data collection.

Results: Statistically significant differences were noted when comparing mean inter-canine and molar widths between pre- and post-treatment periods among extraction cases in Class I malocclusion (p < 0.001). Conversely, there were no significant changes observed in arch widths among non-extraction cases in Class I malocclusion. Similarly, significant changes were observed in both extraction and non-extraction cases of Class II malocclusion when comparing mean inter-canine and molar widths between pre- and post-treatment periods (p < 0.05).

Conclusion: After treatment, both Class I and Class II extraction cases showed an increase in inter-canine arch width, while intermolar arch width remained unchanged, suggesting that the treatment did not significantly alter the buccal corridor. Additionally, there were no notable changes in inter-canine arch widths between pre- and post-treatment in Class I non-extraction cases. However, the Class II non-extraction group exhibited increased upper and lower inter-canine arch widths after treatment.

## Introduction

In contemporary dentistry, timely detection of oral diseases is crucial, with factors like arch width, length, and depth of dental arches holding substantial importance in orthodontics. It's worth noting that dental arches undergo systematic changes during growth, which tend to diminish in adulthood [1]. One of the key goals for orthodontic researchers is to establish a universally ideal arch form. It is widely understood that the supporting bone initially shapes the arch form, followed by the eruption of teeth and the influence of circumoral musculature and intraoral forces. Therefore, during orthodontic treatment, preserving the original arch form is crucial, with a focus on maintaining stability and minimizing the risk of relapse [2]. Indeed, in a diverse population, traditional fixed measurements for dental arches have become increasingly challenging due to the impact of various factors on the growth and development period. As a clinician, it is imperative to acknowledge that treatment is tailored to an individual and not simply a segment of the population [1].

During orthodontic treatment, orthodontists are significantly concerned about the effect of facial profile, whether it involves tooth extraction or not [3]. The decision to perform extractions is typically made by an experienced orthodontist during the treatment planning process. There is a current dispute about whether extraction or non-extraction therapy yields superior long-term results. In definite cases, the decision to perform extractions might be relatively straightforward. However, for cases where either approach could be viable, orthodontists may face a dilemma. In such borderline cases, the decision regarding the treatment approach involves careful consideration of factors such as the impact on the facial profile, long-term stability, and the smile aesthetics of the treatment outcomes. These factors play a critical role in guiding orthodontists in determining the most appropriate treatment option for each patient.

A frequently neglected aspect of orthodontic extraction treatment is its tendency to lead to narrower dental arches compared to non-extraction approaches. This narrowing can impact the buccal corridor, the space visible between the back teeth, and the corner of the mouth during smiling, often resulting in a darker or black appearance. Some argue that this reduced width may create the perception of inadequate tooth display when smiling. However, research conducted by Johnson and Smith et al. introduced the concept of the buccal corridor ratio, demonstrating that smile aesthetics remain consistent regardless of whether extraction or non-extraction treatment is pursued [4].

Recent research comparing changes in buccal corridors following these treatments revealed intriguing results. Extraction patients showed a slight increase in mandibular inter-canine width but a decrease in intermolar width, suggesting a shift in dental arch proportions [5]. This study aimed to further evaluate and compare these width changes in both anterior and posterior regions for Class I and Class II malocclusions.

## Materials and methods

The Ethical Committee of Vokkaligara Sangha Dental College & Hospital, Bangalore, India, issued approval for the study (KIMS/IEC/D017/2019). All subjects gave their informed consent for inclusion before they participated in the study. After obtaining ethical approval, dental casts were collected from patients treated at the Orthodontics and Dentofacial Orthopedics Department, Vokkaligara Sangha Dental College & Hospital, Bangalore, India, ensuring homogeneity and study suitability. The armamentarium for the study included a digital vernier caliper (SAFESEED Electronic Digital Vernier Caliper, 150 mm, SAFESEED, China) and dental casts.

Inclusion criteria were individuals aged 11-35 with permanent dentition and Angle’s Class I and II malocclusions treated with MBT (McLaughlin, Bennett, and Trevisi) 0.022” slot. Exclusions were craniofacial anomalies, gingival/periodontal diseases, medical conditions, and asymmetries. The initial record examination confirmed eligibility with photographs, radiographs, and study models.

Forty patients were selected: Group I with 10 Class I malocclusion patients undergoing orthodontic treatment with and 10 without extraction, and Group II with 20 Class II malocclusion patients, similarly treated.

**Figure 1:**
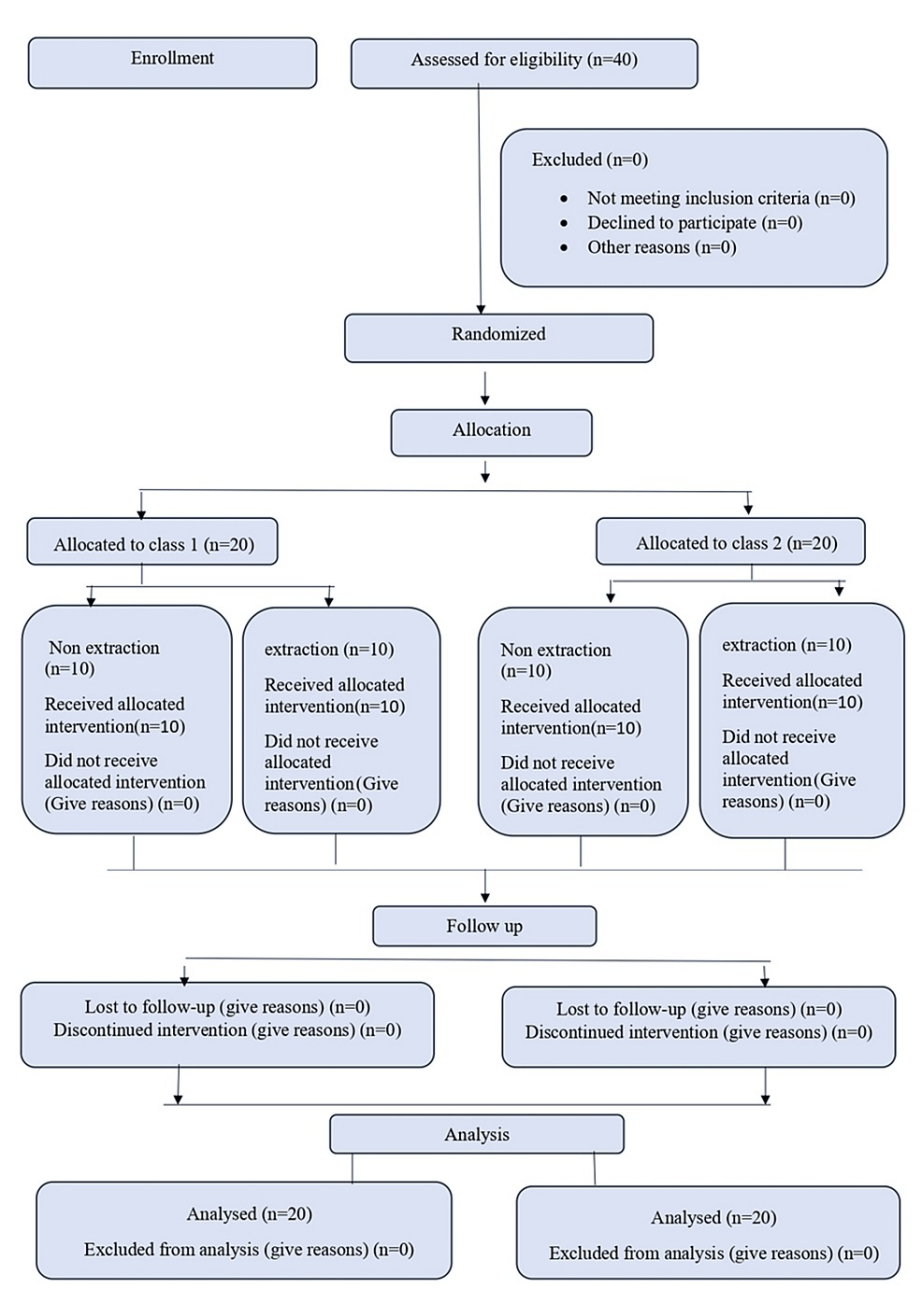
Study methodology represented in the form of a flowchart.

Each patient underwent fixed mechanotherapy utilizing pre-adjusted edgewise appliances with the MBT 0.022’’ slot system in both upper and lower arches. Digital Vernier calipers were employed to measure dental arch widths at the canine and molar regions on pre- and post-treatment study models, ensuring measurement consistency. Figure 1 maps the flowchart explaining the study methodology.

**Figure 2:**
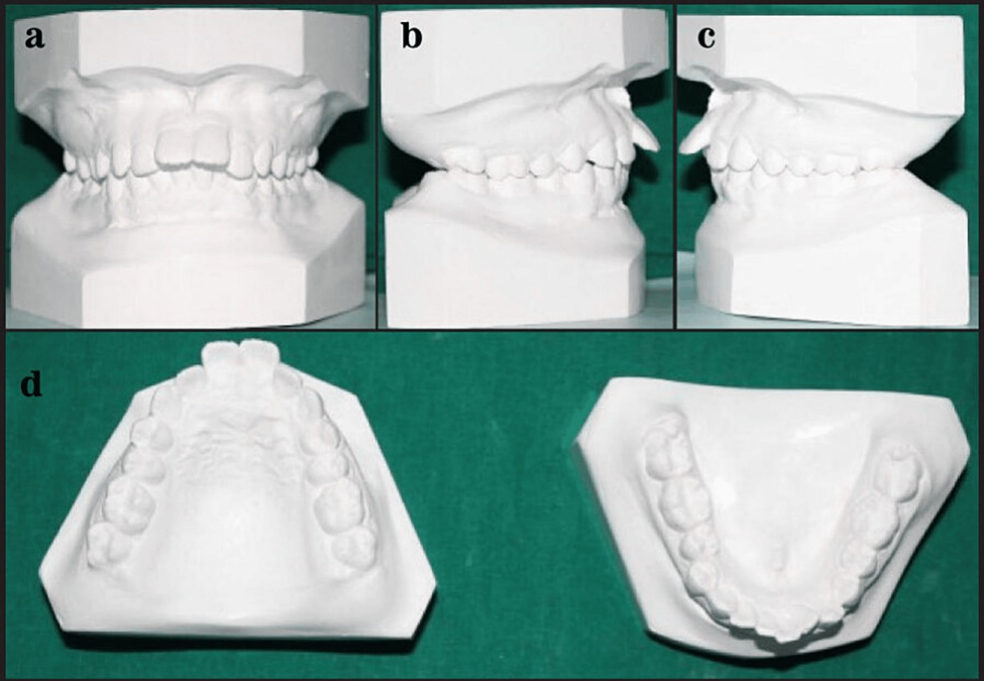
Class I non-extraction study model.

Figure 2 shows the Class I non-extraction study model.

**Figure 3:**
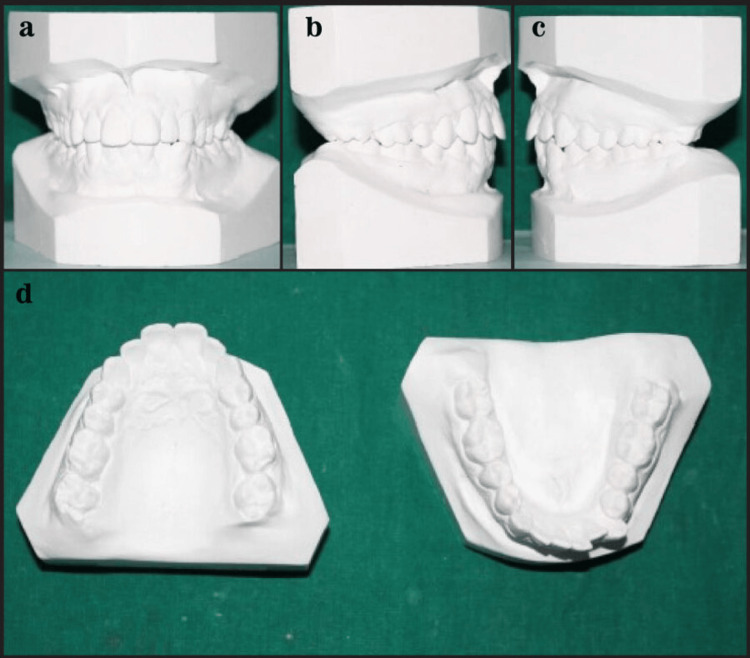
Class I extraction study model.

Figure 3 shows the Class I extraction study model.

**Figure 4:**
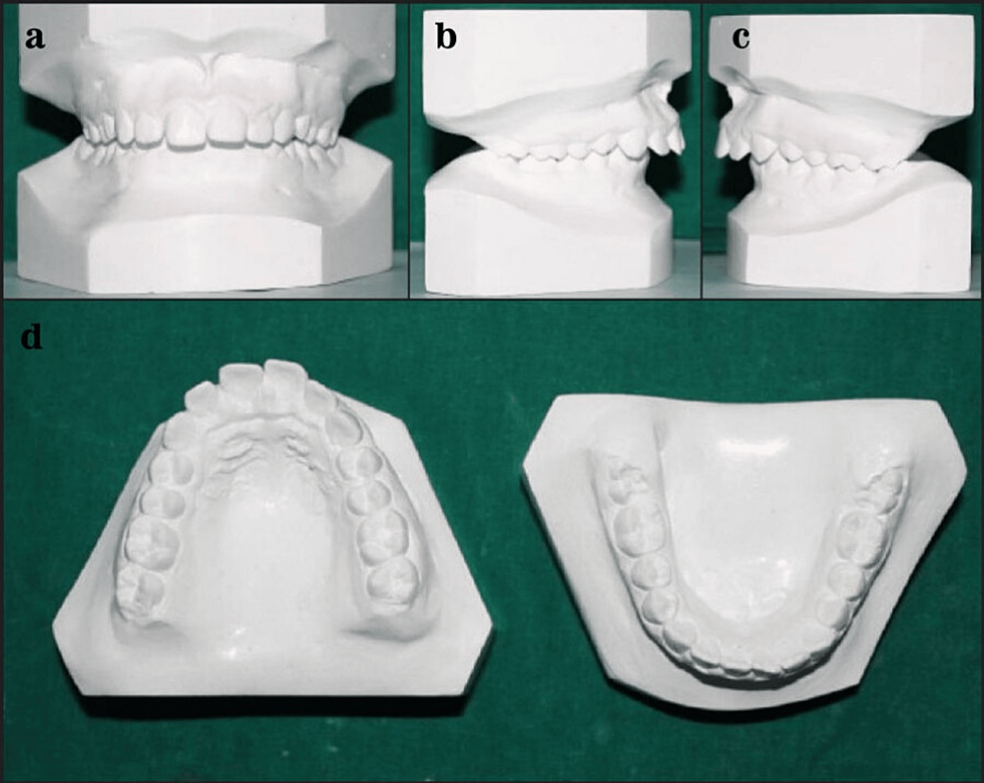
Class II non-extraction study model.

Figure 4 shows the Class II non-extraction study model.

**Figure 5:**
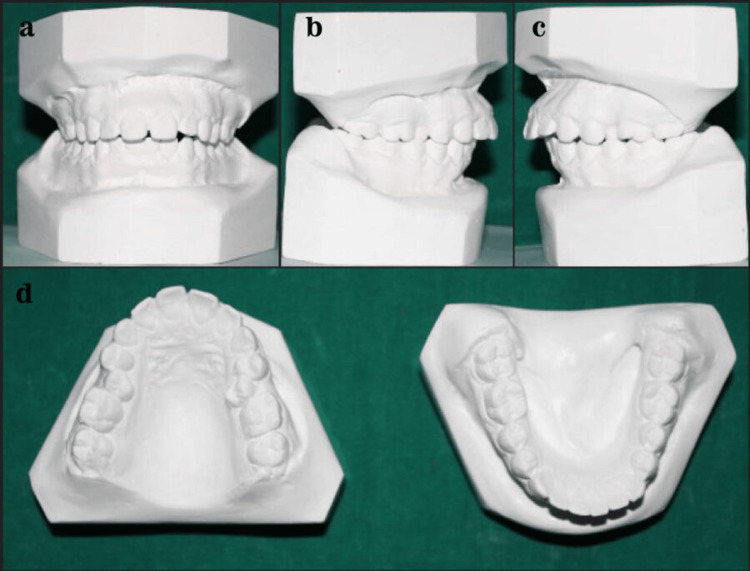
Class II extraction study model.

Figure 5 shows the Class II extraction study model.

Data were organized in Microsoft Excel (Microsoft Corporation, Redmond, Washington, United States), and statistical analysis, including means, standard errors, standard deviations, and significance level t-tests, was conducted using IBM SPSS Statistics for Windows, Version 26, (Released 2019; IBM Corp., Armonk, New York, United States).

## Results

In this study, 40 subjects were involved, with the Class I malocclusion extraction group consisting of 10% males and 90% females, and the non-extraction group consisting of 20% males and 80% females. For Class II malocclusion, the extraction group comprised 60% males and 40% females, while the non-extraction group included 30% males and 70% females.

In this study, for Class I malocclusion, the extraction group included individuals aged 14-25 years, while the non-extraction group comprised individuals aged 11-28 years. Regarding Class II malocclusion, the extraction group consisted of individuals aged 12-24 years, and the non-extraction group encompassed individuals aged 11-19 years.

**Figure 6:**
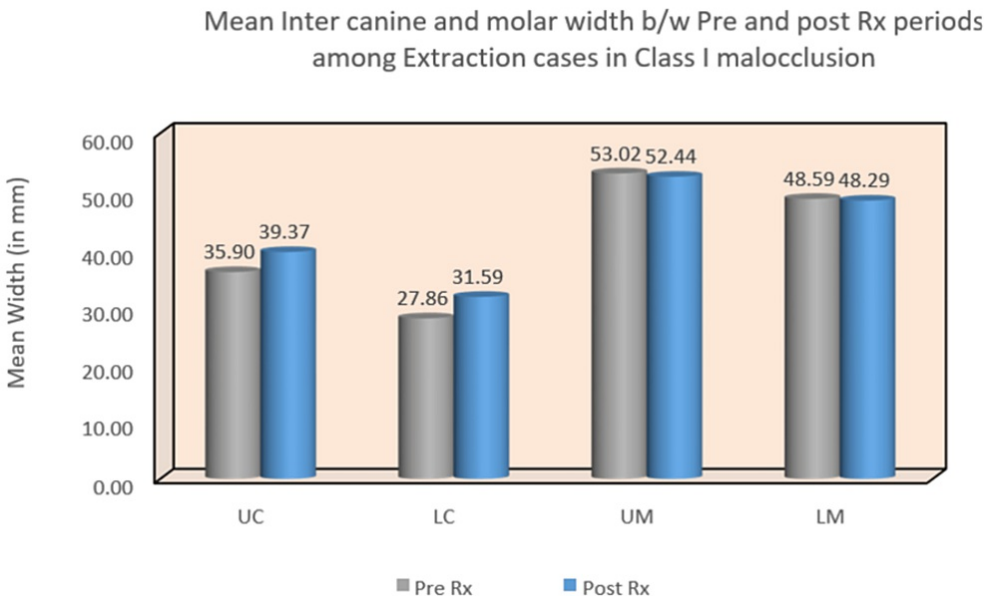
Mean inter-canine and intermolar widths between pre- and post-treatment periods among extraction cases in Class I malocclusion. UC: upper canine; LC: lower canine; UM: upper molar; LM: lower molar

**Figure 7:**
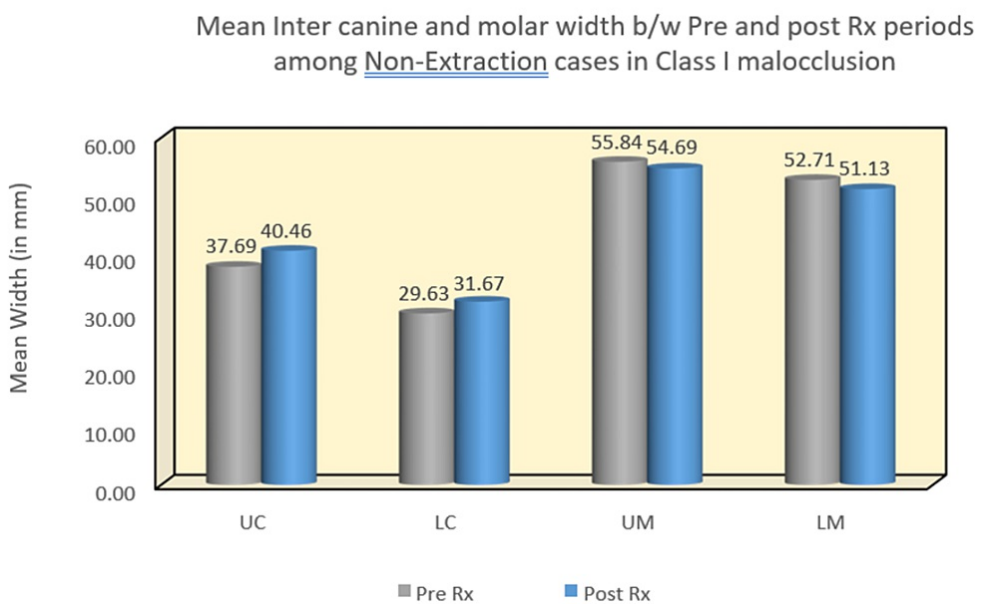
Mean inter-canine and intermolar widths between pre- and post-treatment periods among non-extraction cases in Class I malocclusion. UC: upper canine; LC: lower canine; UM: upper molar; LM: lower molar

In the Class I malocclusion group, significant differences were observed in all four arch width areas. Both upper and lower inter-canine widths showed statistically significant mean differences of -3.16 (p = 0.01) and -2.87 (p = 0.002), respectively. Similarly, the upper and lower intermolar arch widths exhibited statistically significant changes with mean differences of -3.54 (p = 0.008) and -3.97 (p = 0.005), respectively. Conversely, in the Class II malocclusion group, the lower inter-canine and intermolar arch widths displayed statistically significant mean differences of 1.92 (p = 0.04) and 2.80 (p = 0.03), respectively. However, no statistically significant difference was found when comparing the upper inter-canine and upper intermolar arch widths (Table 1) (Figure 6) (Figure 7).

**Table 1:** Comparison of mean inter-canine and intermolar widths (in mm) between extraction and non-extraction cases at pre-treatment and post-treatment periods in Class I and Class II malocclusion. *Indicates that the P-value is significant. For our study, p<0.05 is statistically significant and p<0.001 is highly significant. UC: upper canine; LC: lower canine; UM: upper molar; LM: lower molar

Parameter	Extraction	Non-extraction	Mean difference	p-value	Extraction	Non-extraction	Mean difference	p-value
Class I malocclusion								
	Pre-treatment				Post-treatment			
UC	35.90+1.91	39.06+2.92	-3.16	0.01^*^	39.37+0.87	39.47+1.37	-0.10	0.84
LC	27.86+1.63	30.73+1.85	-2.86	0.002^*^	31.59+0.81	31.55+1.53	0.04	0.95
UM	53.02+2.90	56.57+2.45	3.54	0.008^*^	52.44+1.70	56.78+2.50	-4.34	<0.001^*^
LM	48.59+3.36	52.56+2.07	3.97	0.005^*^	48.29+2.04	52.90+2.62	-4.61	<0.001^*^
Class II malocclusion								
	Pre-treatment				Post-treatment			
UC	37.69+2.75	35.49+2.95	2.20	0.10	40.46+2.22	37.23+2.19	3.24	0.004^*^
LC	29.63+1.74	27.72+2.22	1.92	0.04^*^	31.67+1.85	30.14+1.27	1.53	0.04^*^
UM	55.84+4.26	52.91+2.70	2.94	0.08	54.69+2.85	53.28+2.17	1.41	0.23
LM	52.71+3.05	49.92+2.18	2.80	0.03^*^	51.13+4.86	50.34+2.27	0.78	0.65

In the Class I malocclusion group, there was no significant difference observed in the upper and lower inter-canine arch widths. However, both upper and lower intermolar arch widths demonstrated statistically significant changes, with mean differences of -4.34 (p < 0.001) and -4.61 (p < 0.001), respectively. Conversely, the Class II malocclusion group displayed a statistically significant difference in both upper and lower inter-canine arch widths, with mean differences of 3.24 (p = 0.004) and 1.53 (p = 0.04), respectively. However, there was no statistically significant difference noted in intermolar width.

**Figure 8:**
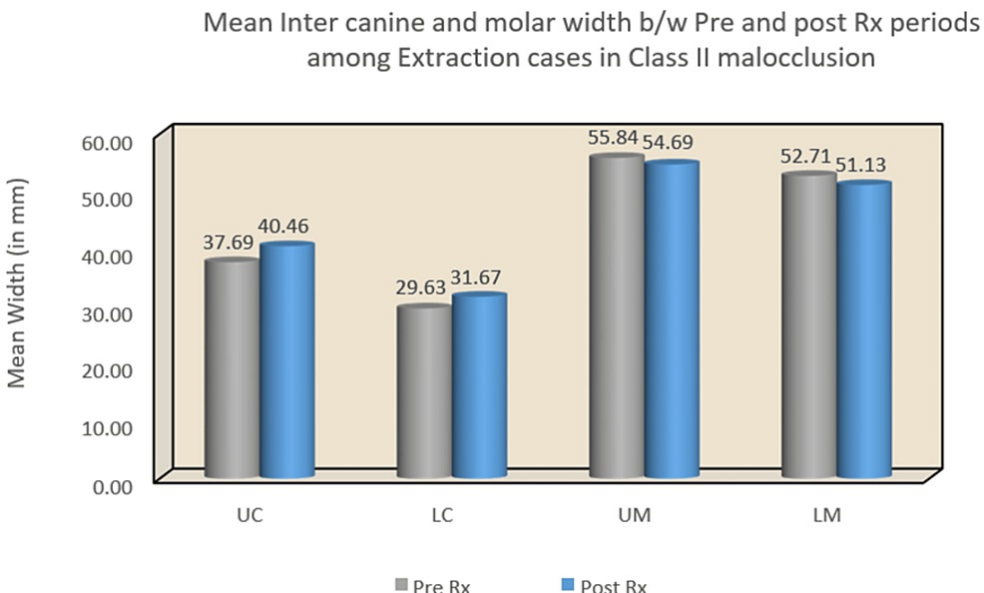
Mean inter-canine and intermolar widths between pre and post-treatment periods among extraction cases in Class II malocclusion. UC: upper canine; LC: lower canine; UM: upper molar; LM: lower molar

**Figure 9:**
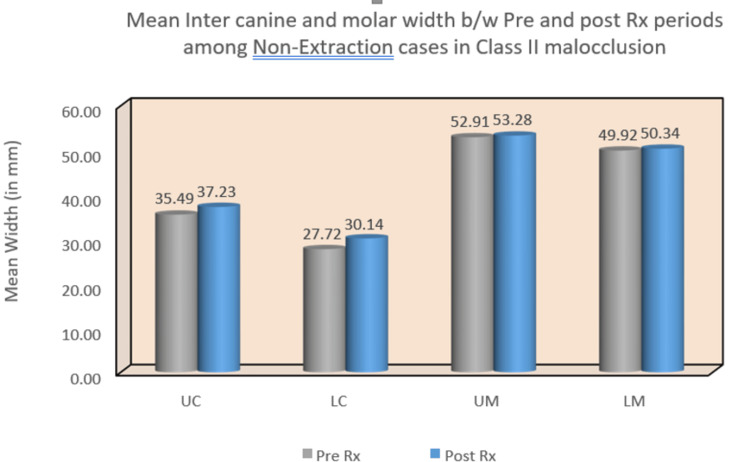
Mean inter-canine and intermolar widths between pre and post-treatment periods among non-extraction cases in Class II malocclusion. UC: upper canine; LC: lower canine; UM: upper molar; LM: lower molar

In the Class I malocclusion group, significant changes were observed in both upper and lower inter-canine arch widths, with mean differences of -3.47 (p < 0.001) and -3.73 (p < 0.001), respectively. However, there were no statistically significant changes detected in upper and lower intermolar arch widths. Conversely, the Class II malocclusion group exhibited significant changes in upper and lower inter-canine arch widths, with mean differences of -2.77 (p = 0.02) and -2.04 (p = 0.003), respectively (Table 2) (Figure 8) (Figure 9).

**Table 2:** Comparison of mean inter-canine and molar widths (in mm) between pre- and post-treatment periods among extraction and non-extraction cases in Class I and Class II malocclusion. *Indicates that the p-value is significant. For our study, p<0.05 is statistically significant, and p<0.001 is highly significant. UC: upper canine; LC: lower canine; UM: upper molar; LM: lower molar

Parameters	Extraction	Extraction	Mean difference	p-value	Non-Extraction	Non-Extraction	Mean difference	p-value
Class I Malocclusion								
	Pre-treatment	Post-treatment			Pre-treatment	Post-treatment		
UC	35.90+1.91	39.37+0.87	-3.47	<0.001^*^	39.06+2.92	39.47+1.37	-0.41	0.66
LC	27.86+1.63	31.59+0.81	-3.73	<0.001^*^	30.73+1.85	31.55+1.53	-0.82	0.24
UM	53.02+2.90	52.44+1.70	0.58	0.47	56.57+2.45	56.78+2.50	-0.22	0.36
LM	48.59+3.36	48.29+2.04	0.30	0.73	52.56+2.07	52.90+2.62	-0.34	0.36
Class II Malocclusion								
	Pre-treatment	Post-treatment			Pre-treatment	Post-treatment		
UC	37.69+2.75	40.46+2.22	-2.77	0.02^*^	35.49+2.95	37.23+2.19	-1.74	0.04^*^
LC	29.63+1.74	31.67+1.85	-2.04	0.003^*^	27.72+2.22	30.14+1.27	-2.42	0.01^*^
UM	55.84+4.26	54.69+2.85	1.15	0.11	52.91+2.70	53.28+2.17	-0.37	0.46
LM	52.71+3.05	51.13+4.86	1.59	0.21	49.92+2.18	50.34+2.27	-0.42	0.58

**Figure 10:**
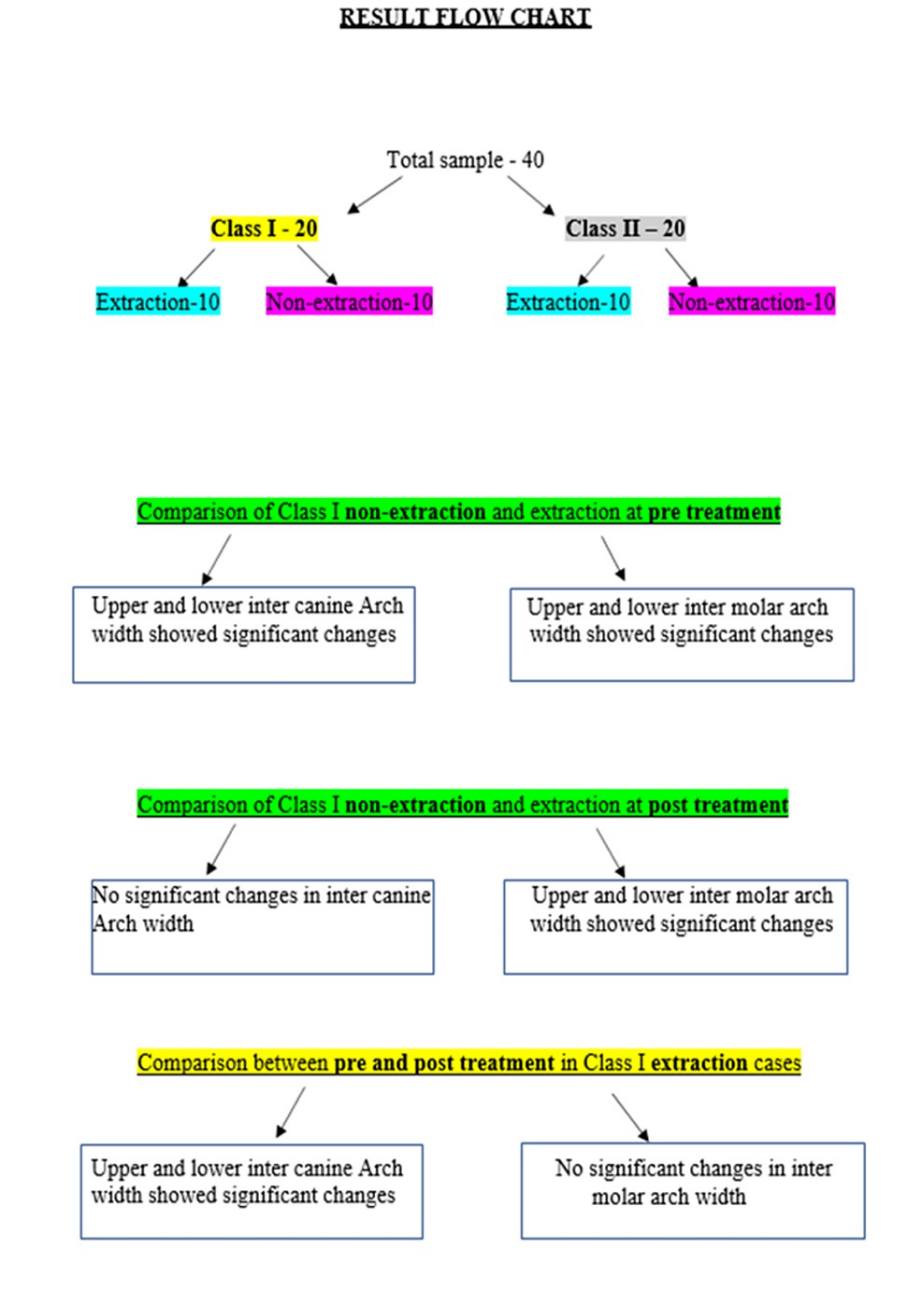
Flowchart summarizing the results of this clinical study.

**Figure 11:**
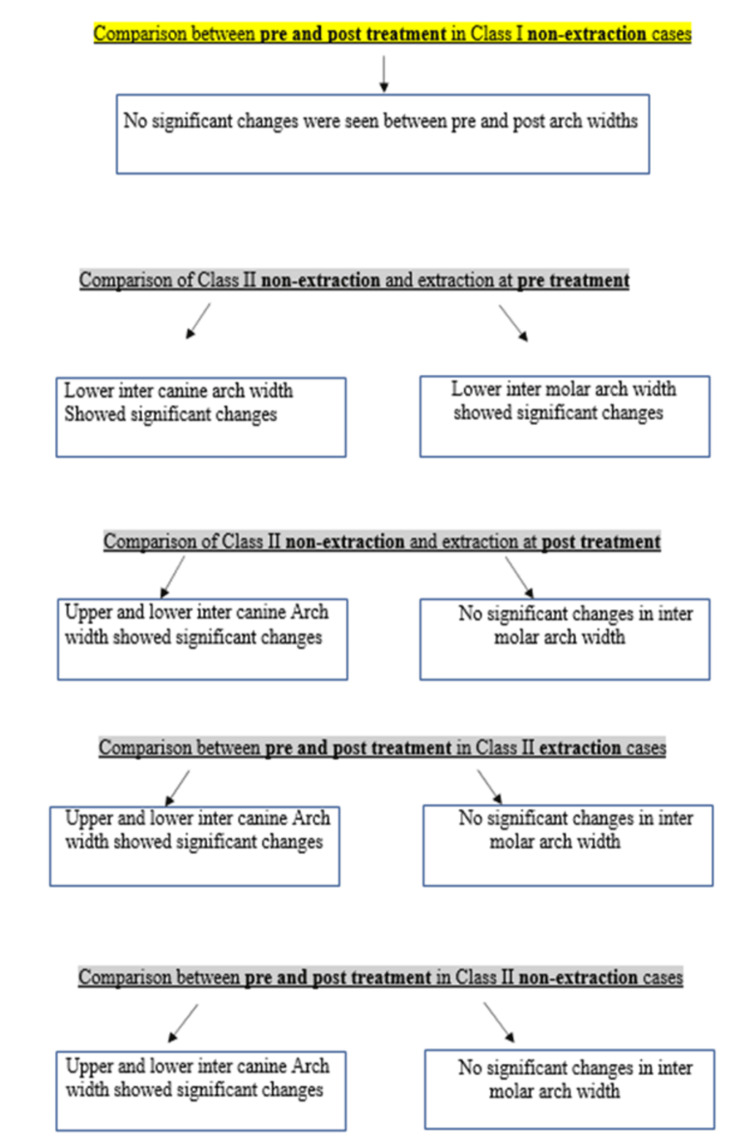
Flowchart summarizing the results of this clinical study. Continued from Figure 10

While the Class I malocclusion group did not demonstrate statistically significant differences in any of the four areas of interest, the Class II malocclusion group showed significant changes in upper and lower inter-canine arch widths, with mean differences of -1.74 (p = 0.04) and -2.42 (p = 0.01), respectively. The summary flowchart of the results is shown in Figure 10 and Figure 11.

## Discussion

Over the past century, orthodontics has seen significant advancements, with professionals continuously refining techniques and appliances. These developments, carefully selected over time, ensure that only the most effective strategies are employed in modern orthodontic practice. Despite notable advancements, doubts persist regarding certain aspects of orthodontic treatment, particularly the effects of premolar extraction on facial profile [6].

It's widely recognized that orthodontic treatment, including tooth extraction, leads to dimensional alterations in dental arches, which endure beyond the active treatment phase. Numerous studies have extensively explored changes in arch width associated with both extraction and non-extraction approaches. However, it's essential to acknowledge potential biases inherent in these studies, which could impact the accuracy of reported changes. Furthermore, variations between treatment groups before the initiation of treatment may obscure genuine treatment effects, potentially attributing outcomes to chance rather than the treatment itself.

Statistical analysis of the study revealed several notable findings. In Class I pre-treatment, the upper canine width increased, while in Class II pre- and post-treatment, both upper and lower inter-canine widths increased. Furthermore, the lower inter-canine width increased in the Class II pre-treatment group. Intermolar width increased in both pre- and post-treatment stages between extraction and non-extraction groups. Additionally, lower intermolar width increased in the Class II pre-treatment group (Table 1).

Moreover, inter-canine width increased between pre- and post-treatment groups in both Class I and Class II extraction cases, as well as in Class II non-extraction cases. However, statistically significant changes in intermolar width were not observed in either extraction or non-extraction cases of Class I and Class II (Table 2). Notably, variations in the width between upper canines can be expected as the width of the lower anterior arch influences the maxillary anterior arch width [7-9]. The observed changes in inter-canine width align with findings from several other studies conducted by Aksu et al. [10], Weinberg and Sadowsky [11], Glenn et al. [5], and Boley et al. [12], all reporting similar trends. Specifically, the increase in arch width changes seen in the study can be attributed to the movement of canines to a more posterior position, resulting in a wider arch plane.

However, our findings differ from a study that observed a decrease in inter-canine width in the non-extraction group [13]. This discrepancy may arise from differences in arch forms used in their study, aiming to retain inter-canine distance at the beginning of treatment.

The study findings, although not reaching statistical significance, do suggest the presence of mild changes in intermolar width. These findings contradict the findings reported in several other studies, which stated a significant increase in intermolar width among non-extraction groups [10, 13-16]. On the other hand, Boley et al. [12], Kim and Gianelly [15], and Gardner and Chaconnas [16] observed a decrease in both upper and lower intermolar widths among extraction groups. Shirazi et al. [17] observed an increase in lower intermolar width among extraction groups.

Several factors could account for the variations in the findings of the study. For instance, utilization of maxillary expansion appliances in those studies, variations in the age groups and gender of the participants, differences in dentition status, and variances in the type of extraction employed in each study. It is worth noting that these factors may have contributed to the divergent outcomes observed in the aforementioned studies.

The present study’s limitations, such as the smaller sample size and the uneven distribution of gender, could impact the accuracy and robustness of the results. To overcome these limitations and to ensure more accurate and reliable findings, future studies should consider enrolling a larger number of subjects that meet the necessary inclusion criteria. Additionally, efforts should be made to achieve a more balanced distribution of gender across the study groups to lessen the impact of gender-related differences on the results to, ultimately, enhance the generalizability and specificity of the outcomes.

## Conclusions

Based on the study's findings, it appears that when extraction is part of orthodontic treatment for both Class I and Class II malocclusions, notable alterations occur in the anterior arch width. However, there were no significant changes observed in the posterior arch width. This suggests that the buccal corridor, a crucial aspect of facial aesthetics, remains unchanged, which is generally considered favorable for overall facial appearance.
